# Supplementary material for: Carrageenan of Red Algae Eucheuma gelatinae: Extraction, Antioxidant Activity, Rheology Characteristics, and Physicochemistry Characterization
Source: Molecules. 2022 Feb 14;27(4):1268. doi: 10.3390/molecules27041268 (PMC8875568; doi:10.3390/molecules27041268)
Supplement: Supplementary file 1 [file molecules-27-01268-s001.zip › molecules-1561216-supplementary.pdf]

Supplementary Materials

Carrageenan of Red Algae *Eucheuma gelatinae*: Extraction, Antioxidant Activity, Rheology Characteristics, and Physicochemistry Characterization

Hoang Thai Ha [1], Dang Xuan Cuong [1,2,3,*], Le Huong Thuy [4,*], Pham Thanh Thuan [5], Dang Thi Thanh Tuyen [6], Vu Thi Mo [2,3] and Dinh Huu Dong [1]

[1] Department of Food Technology, Ho Chi Minh City University of Food Industry, 700000 Ho Chi Minh, Vietnam
[2] Department of Biology, Graduate University of Science and Technology, VAST, 100000 Ha Noi, Vietnam
[3] Department of organic material from marine resource, Nha Trang Institute of Technology Research and Application, VAST, 650000 Khanh Hoa, Vietnam
[4] Institute of Biotechnology and Food Technology, Industrial University of Ho Chi Minh City, 700000 Ho Chi Minh, Vietnam
[5] General Surgery Department, Ninh Thuan Provincial General Hospital, 59000 Ninh Thuan, Vietnam
[6] Department of Food Science, Nha Trang University, 650000 Khanh Hoa, Vietnam

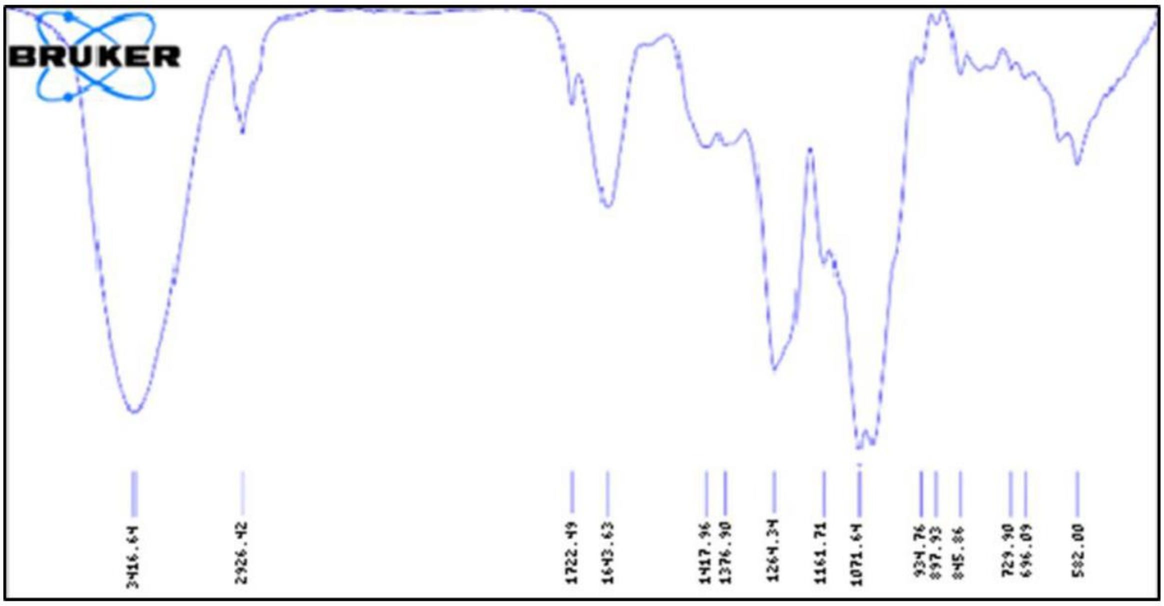

Figure S1. FTIR spectroscopy of antioxidant carrageenan.
